# Fast and High-Accuracy Localization for Three-Dimensional Single-Particle Tracking

**DOI:** 10.1038/srep02462

**Published:** 2013-08-19

**Authors:** Shu-Lin Liu, Jicun Li, Zhi-Ling Zhang, Zhi-Gang Wang, Zhi-Quan Tian, Guo-Ping Wang, Dai-Wen Pang

**Affiliations:** 1 Key Laboratory of Analytical Chemistry for Biology and Medicine (Ministry of Education), College of Chemistry and Molecular Sciences, State Key Laboratory of Virology, and Wuhan Institute of Biotechnology, Wuhan University, Wuhan, 430072, P. R. China; 2 College of Chemistry and Environmental Engineering, Hubei Normal University, Huangshi, 435002, P. R. China; 3 Department of Physics, Wuhan University, Wuhan, 430072, P. R. China; 4 These authors contributed equally to this work.

## Abstract

We report a non-iterative localization algorithm that utilizes the scaling of a three-dimensional (3D) image in the axial direction and focuses on evaluating the radial symmetry center of the scaled image to achieve the desired single-particle localization. Using this approach, we analyzed simulated 3D particle images by wide-field microscopy and confocal microscopy respectively, and the 3D trajectory of quantum dots (QDs)-labeled influenza virus in live cells. Both applications indicate that the method can achieve 3D single-particle localization with a sub-pixel precision and sub-millisecond computation time. The precision is almost the same as that of the iterative nonlinear least-squares 3D Gaussian fitting method, but with two orders of magnitude higher computation speed. This approach can reduce considerably the time and costs for processing the large volume data of 3D images for 3D single-particle tracking, which is especially suited for 3D high-precision single-particle tracking, 3D single-molecule imaging and even new microscopy techniques.

Single-particle tracking (SPT) is a remarkable tool for studying the dynamics of biomolecules in live cells. Two-dimensional (2D) single-particle tracking has been widely used in many biological fields, such as membrane dynamics^1^,^2^,^3^, virus infection mechanism^4^,^5^,^6^,^7^ and intracellular/intercellular transport dynamics of biomolecules^8^,^9^,^10^, which could determine the particle center with a precision of several nanometers by combining with a 2D Gaussian-fitting method^11^,^12^. However, the main limitation is that 2D SPT can only track individual particles in a thin focal plane.

Three-dimensional (3D) single-particle tracking, which captures the full spatiotemporal behavior of the sub-cellular particles, is becoming increasingly important for understanding cellular dynamics in live cells. Many microscopy techniques have been developed for 3D localization in SPT field^13^,^14^,^15^,^16^,^17^, which also attracts researchers' attentions in many single-particle applications^18^,^19^,^20^,^21^,^22^. There are two main types of techniques for tracking individual particles in three dimensions: 3D tracking by post-analyzing the acquired image data, and real-time 3D tracking by feedback approaches^15^. The first approach is beneficial for tracking multiple particles simultaneously, most of which require the modifications on standard microscope and use 2D Gaussian fitting algorithm for achieving super-resolution localization precision, including astigmatic imaging^23^,^24^, multifocal plane^22^, biplane detection^25^, double-helix point spread function^26^,^27^. With the development of sensitive cameras and bright fluorescent tags, gathering a number of 2D images at different *z*-positions is the most straightforward method for 3D single-particle imaging and tracking in live cells, which has become widely used in many commercialized instruments.

However, although several corresponding algorithms have been developed for locating the particle center from a series of 3D image *z*-stacks^28^,^29^,^30^, the frequently-used centroid and Gaussian nonlinear least-squares fitting methods have important drawbacks for 3D SPT. The high-speed centroid method has very low localization precision and high edge sensitivity. The iterative Gaussian fitting methods, which are considered to be the most accurate algorithm up to date, are unfavorably used for 3D SPT, because the large data volumes require considerable time and cost for spatiotemporal and numerical fitting steps. Additionally, initial values should be set for fitting parameters before calculation, which is a very tedious process. This motived us to consider whether an efficient algorithm could be developed for 3D SPT with high computation speed and high precision.

Herein, we present a localization algorithm based on radial symmetry method to solve the challenges of 3D SPT listed above. Although the whole-pixel and sub-pixel localizations using radial symmetry method in two dimensions have been described in previous reports^31^,^32^, such a method for 3D and sub-pixel localization has not been reported so far. We mainly assess the localization accuracy and computation speed of our method based on two applications from theoretical and practical perspective: (i) localization of simulated 3D particle images by wide-field microscopy and confocal microscopy, and (ii) 3D SPT of quantum dots (QDs)-labeled influenza virus in live host cells. We demonstrate that our method determines the 3D particle center with low edge sensitivity and sub-pixel precision, similar to that of the Gaussian nonlinear least-squares fitting method. Without iterative, numerical fitting steps, the computation speed of our method is about two orders of magnitude higher than that of the Gaussian fitting method, and similar to that of the centroid method. These features make our algorithm to be a very competitive method for 3D SPT applications.

## Results

### 3D single-particle localization method

**Figure 1:**
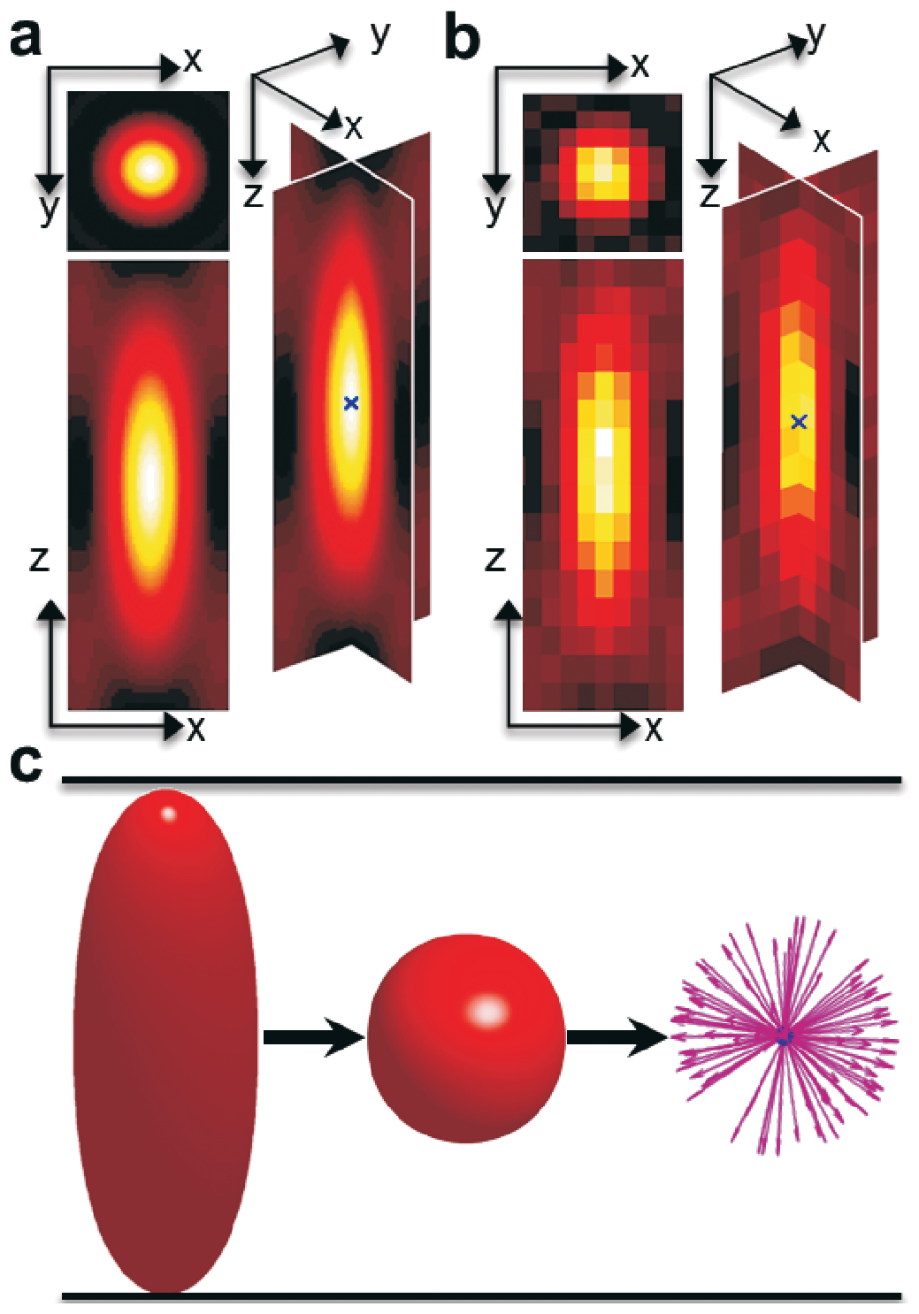
The schematic of the 3D radial symmetry algorithm. (a) The image generated by sampling the point spread function (PSF) of wide-field microscopy on a 3D grid with a lattice size of 20 nm. (b) The 3D CCD image simulated from the PSF image (a) with a signal-to-noise (S/N) ratio of 20. The blue crosses indicate the true center of the 3D particle. (c) The implementation procedure: scaling the 3D image in axial direction, and calculating the center position (the blue point) of the 3D single particle with the minimal distance to all gradient lines (the purple arrow lines).

We introduce the 3D point spread function (PSF) of the Born-Wolf model^33^ to simulate the 3D charge-coupled device (CCD) images of individual particles. For a typical wide-field microscopy, the axial intensity distribution of a 3D PSF is a shape of “butterfly” (Fig. 1a and 1b). The intensity of the “wings” is dramatically reduced for confocal microscopy and the “body” is an approximate ellipsoid, which could be reliably characterized by the ratio of axial resolution (*R_z_*) to lateral resolution (*R_xy_*) (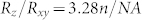, where n is the refractive index of the objective medium and NA is the numerical aperture of the objective.)^34^. Based on this approximation, by scaling 3D images in axial direction according to the ratio, we can obtain the images with the intensity distribution of an approximate sphere. Our 3D radial symmetry localization method utilizes the fact that the gradient line at each pixel in a 3D sphere would intersect theoretically the true center of the 3D sphere, which has a distance of 0 to all gradient lines. Considering any optical noise and deformation error, we estimate the center using the point which has the least total distance to all gradient lines (Fig. 1c). Furthermore, a displacement-weighted method is utilized to decrease the influence of the intensity of the “wings” and makes the approach more accurate in high intensity areas. The arithmetic expressions for calculating the 3D particle center are given explicitly in Supplementary Note.

### Accuracy for wide-field microscopy

**Figure 2:**
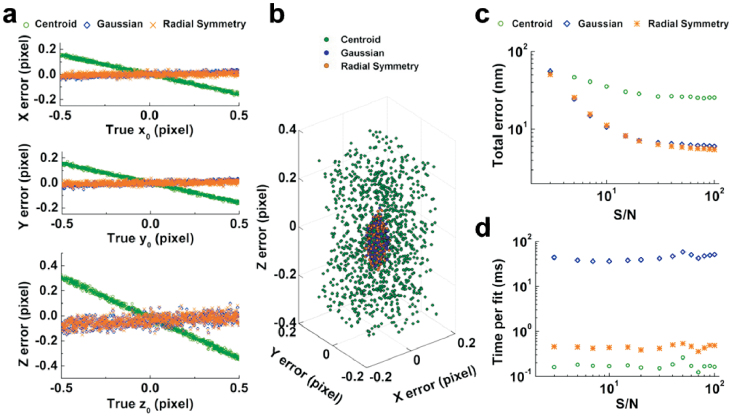
Estimation of the localization accuracy using simulated 3D particle images. (a) Localization errors of 1000 simulated images with the S/N ratio of 20 with centroid, Gaussian fitting, and radial symmetry algorithm, respectively. The errors indicate the difference between the fitting position and the true position in each dimension. (b) The 3D scatter plots of the errors illustrating the error ranges of three methods in three dimensions. (c, d) The total errors and computation times calculated from a series of simulated images with the S/N ratios of 3 ~ 100.

We evaluate the performance of our method for wide-field microscopy by using 1000 3D CCD images, which are simulated with a signal-to-noise (S/N) ratio of 20 and with the true centers distributed randomly between ±0.5 pixels in x, y and z directions. By tracking of 1000 images with a centered 7 × 7 × 11 pixel region (pixel size: 100 × 100 × 150 nm), the 3D radial symmetry method gives a mean lateral error of 0.011 pixel and axial error of 0.044 pixel, which are much smaller than that of centroid method (lateral error: 0.077 pixel, axial error: 0.163 pixel) and very similar to that of Gaussian fitting method (lateral error: 0.011 pixel, axial error: 0.045 pixel) (Fig. 2a). The 3D scatter plots of the errors in three dimensions further illustrate that the error distribution of our method is similar to that of the Gaussian fitting method, and more concentrated than that of the centroid method (Fig. 2b and Supplementary Fig. 1). Meanwhile, we examine the accuracy of the 3D radial symmetry method without scaling the 3D image in axis direction by using simulated 3D CCD images with an S/N ratio of 20. The accuracy of the 3D radial symmetry method (lateral error: 0.018 pixel, axial error: 0.089 pixel) becomes lower than that of the Gaussian fitting method obviously, indicating that the scaling process is very important for improving the precision of the 3D radial symmetry method.

The intensity distribution of the scaled image of a 3D particle (Supplementary Fig. 2a) illustrates that the intensity in axial direction is not only the shape of a sphere, but also contains halos (the “wings” of the 3D PSF image). To examine the influence of the “wings” of the 3D particle image to the localization accuracy, we determine the centers with different lateral sizes. The total error plots show that the accuracies decrease with the shrinking of the lateral size. Furthermore, the accuracy is similar to that of the Gaussian fitting method for large lateral size, and even better for small size (Supplementary Fig. 3a and 3b), indicating that our approach can reduce the surrounding interference.

We further assess the accuracy of our method using simulated 3D images with the S/N ratios of 3 ~ 100. The error plots indicate that the lateral accuracy of our method is slightly lower than that of the Gaussian fitting method for low S/N ratios but higher for high S/N ratios, and the axial accuracy has the same trend (Supplementary Fig. 2b). Furthermore, we calculate the total errors of three localization methods. The total error is 7.06 nm for radial symmetry method, 7.14 nm for the Gaussian fitting method and 28.44 nm for the centroid method at the S/N ratio of 20. The total error plots further suggest that the accuracy of the 3D radial symmetry method is much better than that of the centroid method, and as good as that of the Gaussian fitting method nearly over the entire range, and even better for high S/N ratios (Fig. 2c). Moreover, the average computation time is 0.39 ms for radial symmetry method, 0.16 ms for the centroid method, and 39.12 ms for the Gaussian fitting method, which is about 100 times longer than that of radial symmetry method (Fig. 2d). This is a key attribute of our method for processing a mass of 3D images in SPT field.

### Accuracy for confocal microscopy

As we know, gathering the *z*-stacks by confocal microscopy is a widely-used tool for 3D particle imaging and tracking nowadays. Therefore, we also assess the accuracy of different localization methods using simulated 3D CCD images for confocal microscopy (Supplementary Fig. 4a and 4b). As expected, 3D radial symmetry method (lateral error: 0.009 pixel, axial error: 0.036 pixel, total error: 5.58 nm) is still as accurate as the Gaussian fitting method (lateral error: 0.008 pixel, lateral error: 0.038 pixel, total error: 5.59 nm), and much better than the centroid method (lateral error: 0.064 pixel, axial error: 0.097 pixel, total error: 18.59 nm) for 1000 CCD images with the S/N ratio of 20 (Supplementary Fig. 4c). As above, we examined a series of simulated images with a wide range of S/N values as well, and found that the accuracy of our method is slightly lower than that of the Gaussian fitting method for low S/N ratios but higher in axial direction for high S/N ratios (Supplementary Fig. 5a and 5b). These results demonstrate the fact that the localization accuracy for three methods is improved by using the 3D confocal CCD images and 3D radial symmetry method is applicable for confocal microscopy. Given that the confocal microscope could acquire different number of z slice for 3D particle imaging, we also compared the localization ability of different methods for 3D incomplete images of single particles with different number of z slice. With the decrease of axial size, the accuracy decreases similarly for the three localization methods. But our method is always as accurate as the Gaussian fitting method (Supplementary Fig. 6).

### Accuracy estimated by actual experiments

**Figure 3:**
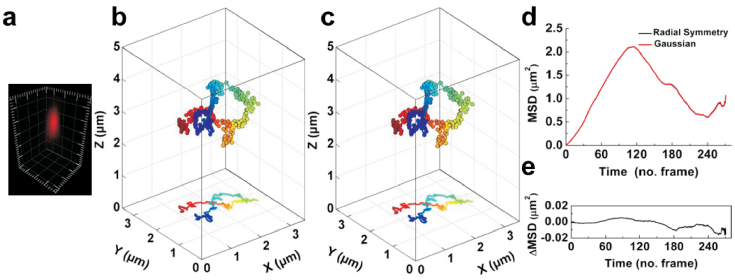
Three-dimensional tracking individual quantum dots-labeled influenza viruses in live cells. (a) 3D images of single virus labeled with QDs for tracking. The length between major tickmark is 1 μm. (b, c) Trajectories of the single virus given by radial symmetry (b) and Gaussian fitting methods (c), respectively. The color of the trajectories indicates a time axis from blue to red. (d) MSD-Time plots of the trajectories analyzed by radial symmetry and Gaussian fitting methods. The difference between the two plots is shown in (e).

To investigate the locating ability under particular distortions or imperfections, we further validated our algorithm using 3D CCD images from actual experiments. We detect the positions of multiple fluorescent beads in 3D images by different methods. The true centers could not be obtained in actual experiments, but the positions located by radial symmetry method are consistent with that of the Gaussian fitting method (Supplementary Fig. 7). Furthermore, we use 3D SPT technique to study the behavior of QDs-labeled influenza virus in live Madin-Darby canine kidney cells and analyze the trajectory of single virus in detail (Fig. 3a). The trajectory of radial symmetry-based tracking is very similar to that of the Gaussian fitting method (Fig. 3b and 3c). It is worthy to point out that the 3D radial symmetry method just spent 0.76 s for calculating the particle positions of the trajectory (273 frames), but 47.77 s was consumed by the Gaussian fitting method. The relationship of mean square displacement (MSD) with time interval is very important for analyzing the motion mode and calculating the relative motional parameters, such as diffusion coefficient and fitting velocity^5^,^35^. We found that the MSD-Time plot for radial symmetry-based tracking is extremely overlapped with that of the Gaussian fitting method (Fig. 3d and 3e). The results further indicate that the radial-symmetry based method is as accuracy as Gaussian fitting method in the actual experiments, and with much higher computation speed.

## Discussion

Although *z*-stacks microscopy has been widely used for 3D single-particle tracking, the relative rapid algorithms for high-resolution single-particle localization remain rarely provided so far. In this work, we developed a non-iterative localization algorithm that first utilizes the scaling of 3D image in the axial direction and then focuses on evaluating the radial symmetry center of the scaled image to achieve the desired single-particle localization. In the first step, the deformation of 3D image of single particles is accomplished by scaling the image in the axial direction based on the ratio of axial resolution to lateral resolution. By evaluating the accuracy using simulated 3D particle images, we confirmed that the scaling step is very essential for improving the accuracy of this localization method. Actually, the ratio of the axial resolution to the lateral resolution may change with different microscopies. The localization method can be adjusted slightly with different microscopies for a higher precision. Localizing the particle position in the second step is based on that particle center has the least total distance to all gradient lines at each pixel in the scaled 3D image. In addition, a displacement-weighted method is supplied in this method to improve the localization precision significantly, which can decrease the influence of the edge intensity. In the end, the combination of these two steps achieves 3D single-particle localization with a sub-pixel localization accuracy and sub-millisecond computation time.

Compared with the frequently-used centroid and Gaussian fitting methods^28^,^29^,^30^, our approach based on the 3D radial symmetry for the 3D single particle localization is more precise than centroid method in the localization precision and has the same precision as the 3D Gaussian fitting method but the computation speed is two orders of magnitude higher than the Gaussian fitting method. The high computation speed and precise localization ability are the main contributions of the 3D radial symmetry method. Meanwhile, although the radial symmetry method has been used in the whole-pixel and sub-pixel localization in two dimensions^31^,^32^, the present 3D radial symmetry method is a significant expansion of the radial symmetry-based algorithm in three dimensions and is expected to be exploited in some new algorithms in the future. Furthermore, by tracking quantum dots (QDs)-labeled influenza virus in live cells, we further confirmed that the 3D radial symmetry method makes it possible to rapidly localize the position of single viruses with a high precision in 3D SPT field. This approach can reduce considerably time and costs for processing the large volume data of 3D images, which is especially suited for the 3D high-precision single-particle tracking, 3D single-molecule imaging and even new microscopy techniques.

## Methods

### Particle localization algorithm

We describe the 3D radial symmetry algorithm in detail in Supplementary Note.

### Simulated 3D particle images

We used the simulated 3D CCD images to assess the precision of the location method. The image was modeled as acquired by a camera, and generated with a 3D PSF of Born-Wolf model^33^. We sampled the PSF on a 3D grid with a lattice size of 20 nm (0.2 pixel in lateral direction and about 0.13 pixel in the axial direction). The smaller lattice size is more close to the real situation, and the localization methods are more accurate. The PSF image was generated randomly following the moving of the centers (*x_0_*, *y_0_*, *z_0_*). The charge-coupled device (CCD) image was simulated by averaging the intensity of the PSF image corresponding to a 7 × 7 × 11 pixel array of the size 100 nm × 100 nm × 150 nm. The pixel size of z is set by the Nyquist formula. To obtain the images with the desired S/N ratio, we normalized the image intensity and then multiplied the (S/N)^2^, so that the peak intensity is equal to (S/N)^2^. Additionally, we generated a Poisson-distribution noise to each pixel with the mean background intensity of ten photons.

### Estimating accuracy

To assess the accuracy of the localization method, we simulate 1000 3D CCD images of individual particles with true centers distributed over the range ± 0.5 pixel (50 nm in lateral direction and 75 nm in axis direction) in each dimension and estimate the precision for each set of parameters.

The error in each dimension is given by 
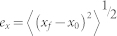

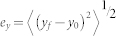

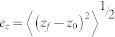


where *e_x_*, *e_y_* and *e_z_* are the errors in x, y and z dimensions, respectively. (*x_0_*, *y_0_*, *z_0_*) is the true center of the particle. (*x_f_*, *y_f_*, *z_f_*) is the particle center determined by the localization methods. The angle brackets indicate averages. The total error (*e_total_*) is obtained by 
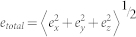


### 3D Gaussian nonlinear least-squares fitting method

The center of 3D CCD image of a particle can be localized by fitting the 3D particle image to a three-dimensional Gaussian function 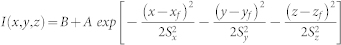
where (*x_f_*_,_ *y_f_*, *z_f_*) is the center of the point source, *A* and *B* are the amplitude and offset, and *S_x_*, *S_y_* and *S_z_* are the width in the x, y and z directions.

### Centroid method

The x position of the particle center can be calculated rapidly by centroid method as 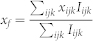


The y and z positions are calculated analogously.

### Imaging of fluorescent beads

We put a drop (5 μL) of suspension containing fluorescent beads with a diameter of 173 nm (505/515, Invitrogen) onto a microscope slide to air dry. Then a small drop of the mounting medium is added and a coverslip was placed to cover the dried spot on the slide. We used a spinning-disk confocal microscope equipped with a Nipkow disk type confocal unit (CSU 22, Yokogawa), a Nano Z Stage (Prior) and an EMCCD (Andor iXon DU885K) for 3D imaging. The fluorescence of beads was excited by a 488 nm laser and filtered with an emission filter of 525/50 nm.

### Tracking quantum dots-labeled influenza virus

We obtained avian influenza A virus (H9N2) strain and label the viruses with quantum dots (QDs) as previous reports. In brief, we produced the influenza virus in the allantoic cavity of 10 day old embryonated eggs. The viruses were biotinylated with Sulfo-NHS-LC-Biotin (Thermo), and then added to the cell surfaces^36^. Subsequently, the cells were incubated with streptavidin-modified 605 nm QDs. The fluorescence of QDs was excited by a 561 nm laser and filtered with an emission filter of 605/20 nm.

## Author Contributions

S.L.L. and J.L. designed and implemented the algorithm; S.L.L. performed the experiments on three-dimensional single-particle tracking; Z.L.Z. discussed the results and commented on the manuscript; Z.G.W. helped analyze the data and discussed the results; Z.Q.T. and G.P.W. discussed the results and commented on the manuscript; D.W.P. initiated the study, discussed the results and commented on the manuscript.

## Supplementary Material

Supplementary Information Supporting Information
