# Supplementary material for: Fast and High-Accuracy Localization for Three-Dimensional Single-Particle Tracking
Source: Sci Rep. 2013 Aug 19;3:2462. doi: 10.1038/srep02462 (PMC3746204; doi:10.1038/srep02462)
Supplement: Supplementary Information — Supporting Information [file srep02462-s1.pdf]

Fast and High-Accuracy Localization for Three-Dimensional Single-Particle Tracking

Shu-Lin Liu, Jicun Li, Zhi-Ling Zhang, Zhi-Gang Wang, Zhi-Quan Tian, Guo-Ping Wang, Dai-Wen Pang*

**Supplementary File**	**Title**
**Supplementary Figure 1**	3D scatter plots of the errors illustrating the error ranges of different algorithms in three dimensions.
**Supplementary Figure 2**	Localization accuracy for different algorithms estimated using simulated 3D CCD images of wide-field microscope.
**Supplementary Figure 3**	Influence of the lateral size on the accuracy for different localization algorithms.
**Supplementary Figure 4**	Localization accuracy for different algorithms estimated using simulated 3D images of confocal microscope.
**Supplementary Figure 5**	Accuracy for different localization algorithms estimated using simulated 3D CCD images of confocal microscope with the S/N ratios of 3 ~ 100.
**Supplementary Figure 6**	Influence of the axial size on the accuracy for different localization algorithms.
**Supplementary Figure 7**	Comparation of the precision for radial symmetry and Gaussian fitting method estimated using 3D confocal images of fluorescence beads.
**Supplementary Note**	3D radial symmetry localization algorithm

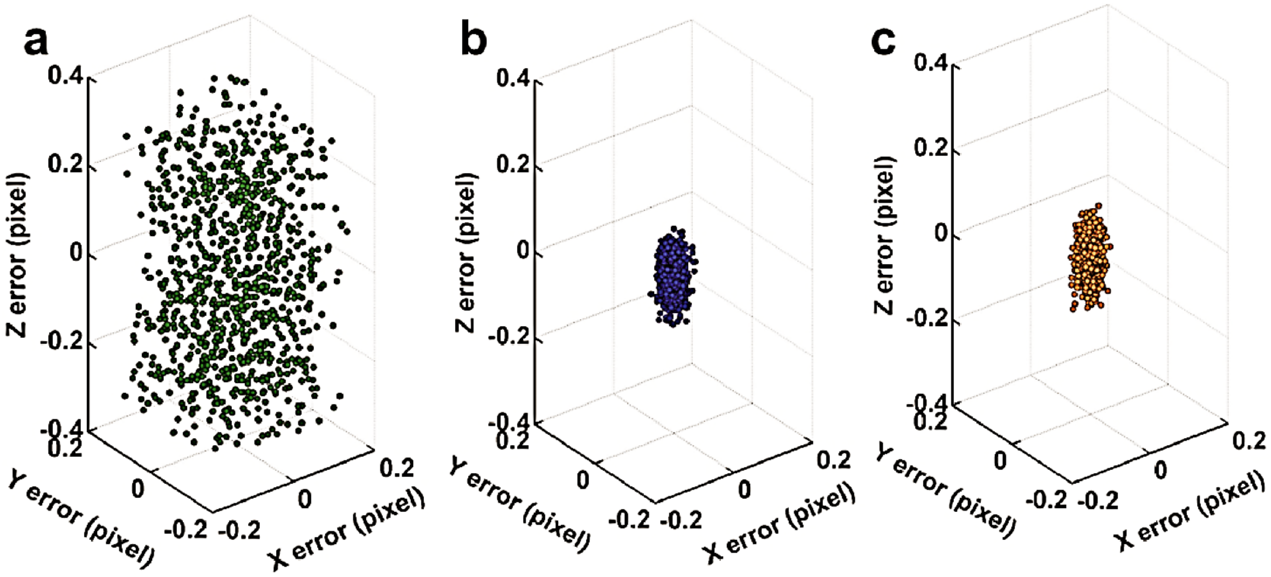

Supplementary Figure 1. 3D scatter plots of the errors illustrating the error ranges of the centroid (a), Gaussian fitting (b), and radial symmetry (c) methods in three dimensions, respectively.

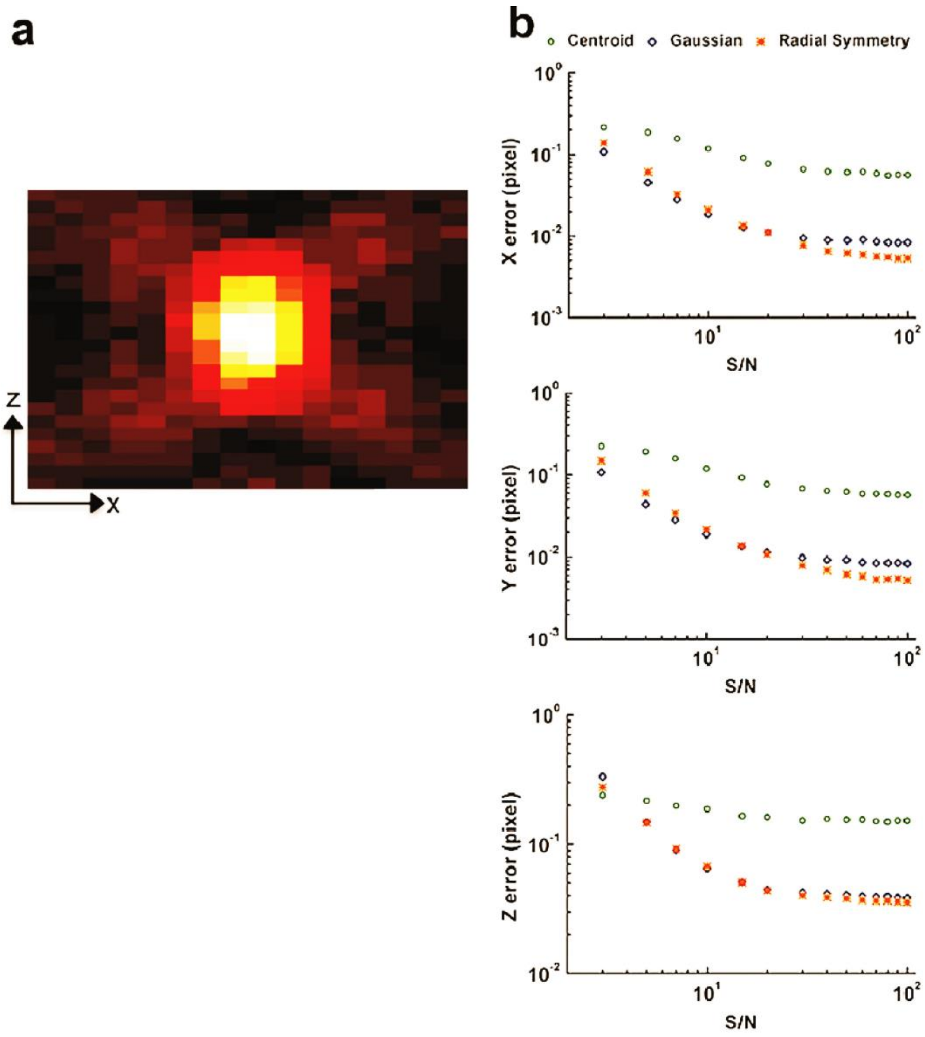

Supplementary Figure 2. Localization accuracy for different algorithms estimated using simulated 3D CCD images of wide-field microscope. (**a**) The x-z plane of the 3D image scaled in axial direction with an S/N ratio of 20. (**b**) Localization errors of different algorithms in each dimension from a series of simulated images with a range of 3 ~ 100 S/N ratios.

.

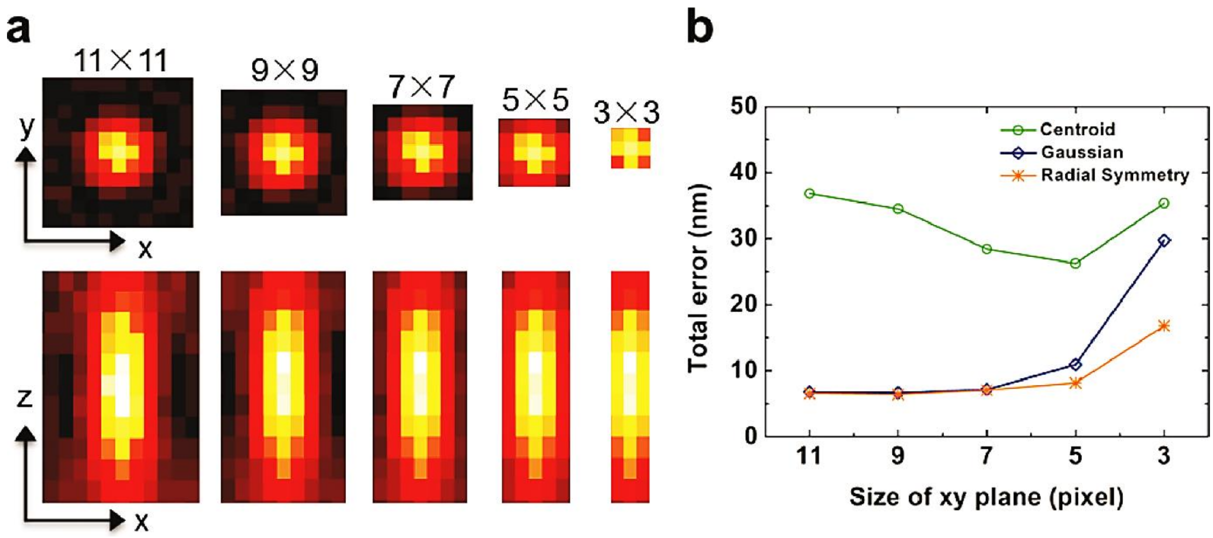

Supplementary Figure 3. Influence of the lateral size on the accuracy for different localization algorithms. 1000 simulated 3D CCD images of wide-field microscope with an S/N ratio of 20 are used. (**a**) The shrink of the lateral size of 3D image. (**b**) The dependence of total error on the lateral size.

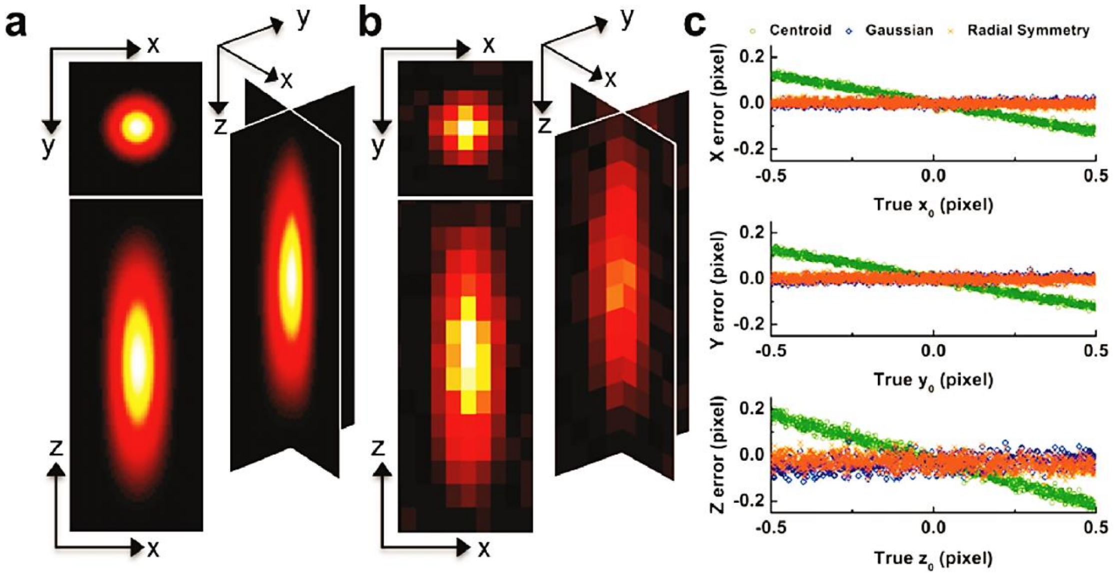

Supplementary Figure 4. Localization accuracy for different algorithms estimated using simulated 3D images of confocal microscope. (**a**) The image generated by sampling point spread function (PSF) on a 3D grid with a lattice size of 20 nm. (**b**) The 3D CCD image simulated from the PSF image (**a**) with an S/N ratio of 20. (**c**) Localization errors of 1000 simulated CCD images with the S/N ratio of 20 localized with centroid, Gaussian fitting, and radial symmetry algorithm, respectively.

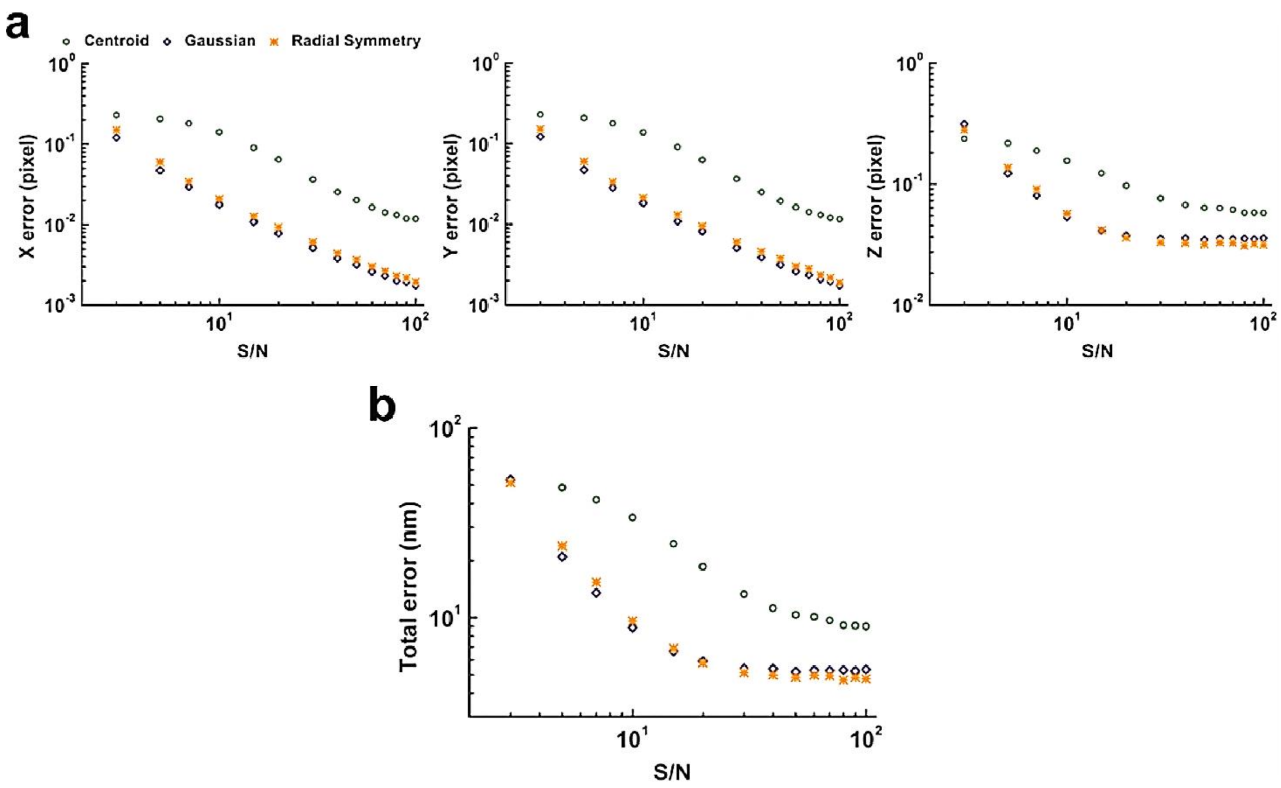

Supplementary Figure 5. Accuracy for different localization algorithms estimated using simulated 3D CCD images of confocal microscope with the S/N ratios of 3 ~ 100. (**a**) The errors of different algorithms in each dimension from a series of simulated images. (b) The total errors of the different algorithms from a series of simulated images.

.

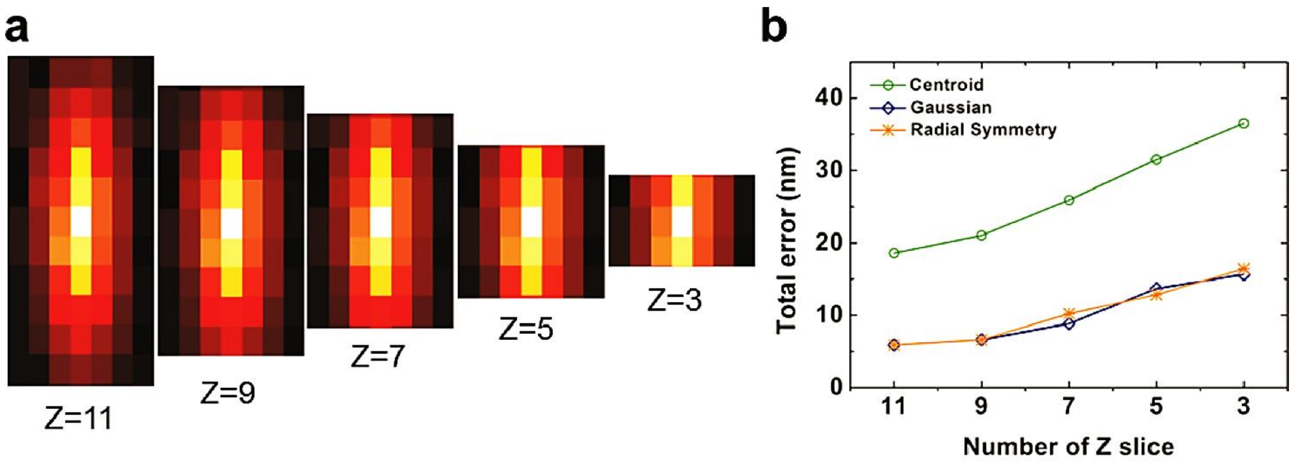

Supplementary Figure 6. Influence of the axial size on the accuracy for different localization algorithms. 1000 simulated 3D CCD images of confocal microscope with an S/N ratio of 20 are utilized. (**a**) The shrink of the axial size in 3D image. (**b**) The dependence of total error on the axial size.

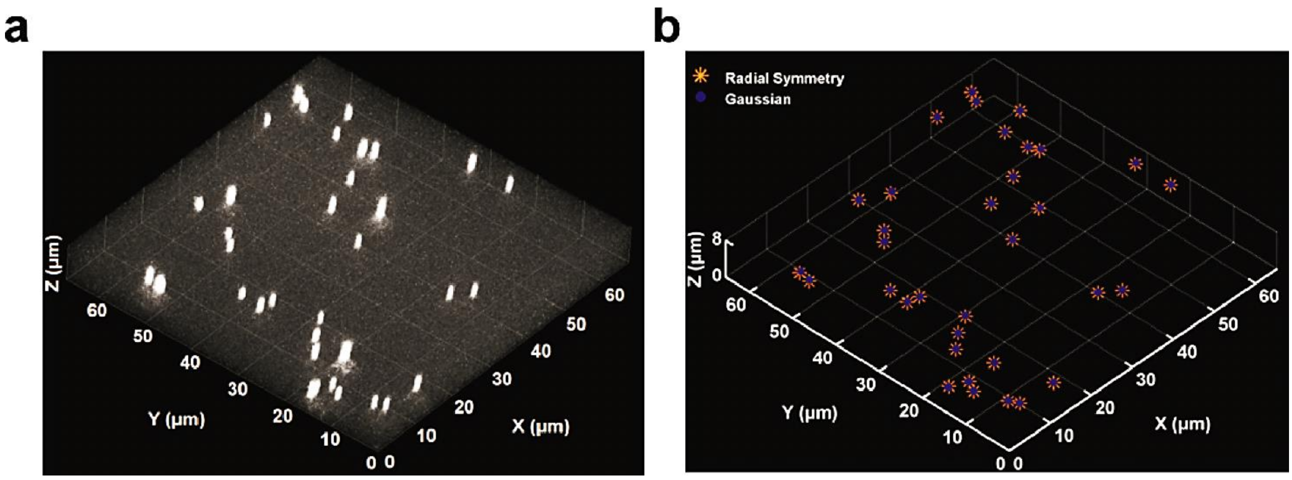

Supplementary Figure 7. Comparation of the precision for radial symmetry and Gaussian fitting method estimated using 3D confocal images of fluorescence beads (**a**). (**b**) The positions of the beads localized by radial symmetry and Gaussian fitting method, respectively.

Supplementary Note: 3D radial symmetry localization algorithm

Three-dimensional (3D) diffraction pattern of a single particle is described as a 3D point spread function (PSF) of Born-Wolf model.

$$PSF(x,y,z) = -\frac{2\pi i a^2 A}{\lambda f^2} e^{i\left(\frac{f}{a}\right)^2 u} \int_0^1 e^{-\frac{iu\rho^2}{2}} J_0(v\rho)\rho d\rho$$

where $u = \frac{2\pi}{\lambda}\left(\frac{a}{f}\right)^2 z$, $v = \frac{2\pi}{\lambda}\left(\frac{a}{f}\right) r$, $r = \sqrt{x^2+y^2}$, $a/f = NA/n$ and $\rho = r/a$. $J_0(v\rho)$ is the Bessel function of zero order. r, a and f are the radial co-ordinate, the radius of the exit pupil and the focal distance of the objective, respectively. λ is the wavelength of light, n is the refractive index of the object medium and NA is the numerical aperture of the objective. A is the amplitude factor.

The intensity distribution of 3D diffraction pattern is described as

$$I(x,y,z) = |PSF(x,y,z)|^{2N+2}$$

where N is 0 and 1for wide-field microscope and for confocal microscope, respectively.

At the geometrical focus, $u = v = 0$ and the intensity is

$$I_0 = \left[\frac{1}{4}\left(\frac{ka^2 A}{f^2}\right)\right]^{N+1}$$

For the points in the focal plane, $u = 0$ and the intensity distribution is

$$I_{xy} = I_0 \left(2\frac{J_1(v)}{v}\right)^{2N+2}$$

The radius of the first dark ring in the focus of 3D diffraction pattern can be calculated from

$$I_{xy} = 0$$

so

$$J_1(v) = 0$$

Thus,

$$v = \frac{2\pi}{\lambda}\left(\frac{a}{f}\right)\sqrt{x^2+y^2} = 3.83$$

$$R_{xy} = \sqrt{x^2+y^2} \approx 0.61\frac{\lambda n}{NA}$$

where R_{xy} is also considered as the lateral resolution according to Rayleigh criteria.

Likewise, for the points along the axis, $v = 0$, and the intensity is expressed as

$$I_z = I_0 \left(\frac{\sin(u/4)}{u/4}\right)^{2N+2}$$

The radius of the first dark ring along the axis of 3D diffraction pattern can be calculated from

$$I_z = 0$$

so that

$$u = \frac{2\pi}{\lambda}\left(\frac{a}{f}\right)^2 z = 4\pi$$

Hence,

$$R_z = \frac{2\lambda n^2}{NA^2}$$

where R_z is also given as the axial resolution.

The ratio of the axial to lateral radius of the 3D diffraction pattern is approximately given as

$$\frac{R_z}{R_{xy}} = \frac{3.28n}{NA}$$

This formula is suitable for both wide-field and confocal microscopy.

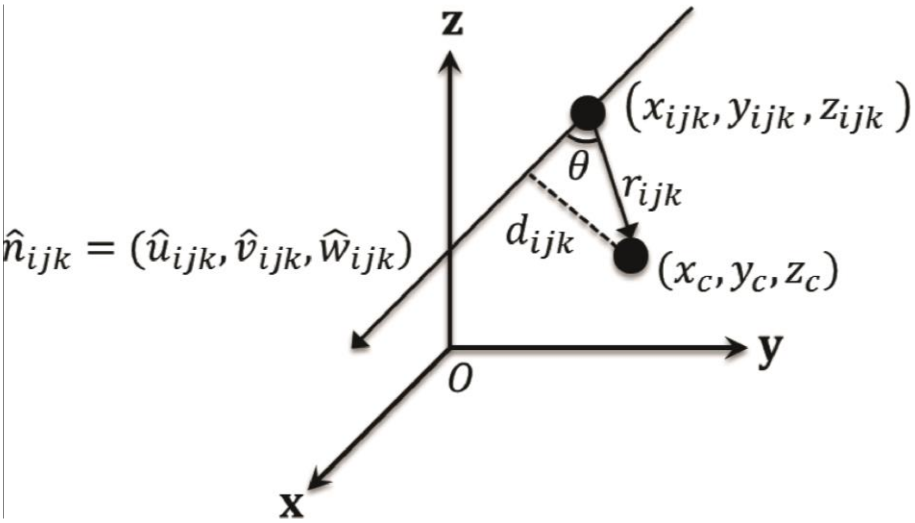

Supplementary Figure 8. Illustration of the calculation of the 3D radial symmetry algorithm.

Therefore, we scale the 3D image of a single particle in the axial direction according to the ratio of the axial and lateral resolution. Considering the estimate error and noise, our algorithm determines the particle center (x_c, y_c, z_c) as the point having the minimal distance to all intensity gradient lines (**Supplementary Figure 8**). The distance (d_{ijk}) of the center to each gradient line can be calculated as follows:

$$d_{ijk}{}^2 = \left|\vec{r}_{ijk}\right|^2 - \left(\left|\vec{r}_{ijk}\right|\cos\theta\right)^2$$

$$\left|\vec{\mathrm{r}}_{ijk}\right|\cos\theta = \vec{r}_{ijk} \cdot \hat{n}_{ijk}$$

where $\hat{n}_{ijk}$ is the unit vector along the gradient line through the point $(x_{ijk}, y_{ijk}, z_{ijk})$, which can be expressed as $(\hat{u}_{ijk}, \hat{v}_{ijk}, \hat{w}_{ijk})$ in three dimensions. Given the intensity (I_{ijk}) at the pixel$(x_{ijk}, y_{ijk}, z_{ijk})$, the vector $(\hat{u}_{ijk})$ in x direction can be estimated as $\hat{u}_{ijk} = \frac{I_{i,j+1,k} - I_{i,j-1,k}}{2d_x\left|\vec{\nabla}I_{ijk}\right|}$, where d_x is the pixel size in x direction, and $\vec{\nabla}I_{ijk}$ is the

gradient magnitude at the point $(x_{ijk}, y_{ijk}, z_{ijk})$, similarly for $\hat{v}_{ijk}$ and $\hat{w}_{ijk}$ in y and z directions. Thus, the distance can be expressed as

$$d_{ijk}^2 = (x_c - x_{ijk})^2 + (y_c - y_{ijk})^2 + (z_c - z_{ijk})^2 - [\hat{u}_{ijk}(x_c - x_{ijk}) + \hat{v}_{ijk}(y_c - y_{ijk}) + \hat{w}_{ijk}(z_c - z_{ijk})]^2.$$

To calculate the center, we minimize $\chi^2 = \sum_{ijk} d_{ijk}{}^2 q_{ijk}$, where q_{ijk} , a displacement weighting, is the square of the gradient magnitude divided by the distance between the pixel $(x_{ijk}, y_{ijk}, z_{ijk})$ and the particle center evaluated using centroid method. We get the derivative of χ^2 with respect to x_c and set it equal to zero, similarly for y_c and z_c respectively. The equations are as follows:

$$x_c \sum_{ijk} q_{ijk}(1 - \hat{u}_{ijk}{}^2) - y_c \sum_{ijk} q_{ijk}\hat{u}_{ijk}\hat{v}_{ijk} - z_c \sum_{ijk} q_{ijk}\hat{u}_{ijk}\hat{w}_{ijk} = -\sum_{ijk}[(\hat{u}_{ijk}{}^2 - 1)x_{ijk}q_{ijk} + q_{ijk}\hat{u}_{ijk}\hat{v}_{ijk}y_{ijk} + q_{ijk}\hat{u}_{ijk}\hat{w}_{ijk}z_{ijk}]$$

$$-x_c \sum_{ijk} q_{ijk}\hat{u}_{ijk}\hat{v}_{ijk} + y_c \sum_{ijk} q_{ijk}(1 - \hat{v}_{ijk}{}^2) - z_c \sum_{ijk} q_{ijk}\hat{v}_{ijk}\hat{w}_{ijk} = -\sum_{ijk}[\hat{u}_{ijk}\hat{v}_{ijk}x_{ijk}q_{ijk} + (\hat{v}_{ijk}{}^2 - 1)y_{ijk}q_{ijk} + q_{ijk}\hat{v}_{ijk}\hat{w}_{ijk}z_{ijk}]$$

$$-x_c \sum_{ijk} \hat{u}_{ijk}\hat{w}_{ijk}\, q_{ijk} - y_c \sum_{ijk} \hat{v}_{ijk}\hat{w}_{ijk}\, q_{ijk} + z_c \sum_{ijk} q_{ijk}(1 - \hat{w}_{ijk}{}^2) = -\sum_{ijk}[\hat{u}_{ijk}\hat{w}_{ijk}x_{ijk}q_{ijk} + \hat{v}_{ijk}\hat{w}_{ijk}y_{ijk}q_{ijk} + (\hat{w}_{ijk}{}^2 - 1)z_{ijk}q_{ijk}]$$

After some mathematical deformation, we solve the following matrix equation to obtain the center (x_c, y_c, z_c).

$$\begin{bmatrix} \sum_{ijk} q_{ijk}(1 - \hat{u}_{ijk}{}^2) & -\sum_{ijk} q_{ijk}\hat{u}_{ijk}\hat{v}_{ijk} & -\sum_{ijk} q_{ijk}\hat{u}_{ijk}\hat{w}_{ijk} \\ -\sum_{ijk} q_{ijk}\hat{u}_{ijk}\hat{v}_{ijk} & \sum_{ijk} q_{ijk}(1 - \hat{v}_{ijk}{}^2) & -\sum_{ijk} q_{ijk}\hat{v}_{ijk}\hat{w}_{ijk} \\ -\sum_{ijk} q_{ijk}\hat{u}_{ijk}\hat{w}_{ijk} & -\sum_{ijk} q_{ijk}\hat{v}_{ijk}\hat{w}_{ijk} & \sum_{ijk} q_{ijk}(1 - \hat{w}_{ijk}{}^2) \end{bmatrix} \begin{bmatrix} x_c \\ y_c \\ z_c \end{bmatrix} = \begin{bmatrix} -\sum_{ijk}[q_{ijk}(\hat{u}_{ijk}{}^2 - 1)x_{ijk} + q_{ijk}\hat{u}_{ijk}\hat{v}_{ijk}y_{ijk} + q_{ijk}\hat{u}_{ijk}\hat{w}_{ijk}z_{ijk}] \\ -\sum_{ijk}[q_{ijk}\hat{u}_{ijk}\hat{v}_{ijk}x_{ijk} + q_{ijk}(\hat{v}_{ijk}{}^2 - 1)y_{ijk} + q_{ijk}\hat{v}_{ijk}\hat{w}_{ijk}z_{ijk}] \\ -\sum_{ijk}[q_{ijk}\hat{u}_{ijk}\hat{w}_{ijk}x_{ijk} + q_{ijk}\hat{v}_{ijk}\hat{w}_{ijk}y_{ijk} + q_{ijk}(\hat{w}_{ijk}{}^2 - 1)z_{ijk}] \end{bmatrix}$$
